# Synthesis of colloidal solutions with silicon nanocrystals from porous silicon

**DOI:** 10.1186/1556-276X-9-571

**Published:** 2014-10-13

**Authors:** José Alberto Luna López, Abel Garzón Román, Estela Gómez Barojas, JF Flores Gracia, Javier Martínez Juárez, Jesús Carrillo López

**Affiliations:** 1 Instituto de Ciencias, Centro de Investigaciones en Dispositivos Semiconductores, Benemérita Universidad Autónoma de Puebla, Ed. 103C o D, Ciudad Universitaria, Col. San Manuel, Puebla, Puebla C.P. 72570, México

**Keywords:** Colloidal solution, Silicon nanocrystal, XRD, PL, FTIR

## Abstract

In this work, we have obtained colloidal solutions of Si nanocrystals (Si-ncs), starting from free-standing porous silicon (PSi) layers. PSi layers were synthesized using a two-electrode Teflon electrochemical cell; the etching solution contained hydrogen peroxide 30%, hydrofluoric acid 40% (HF), and methanol. The anodizing current density was varied to 250 mA cm^-2^, 1 A cm^-2^, and 1.2 A cm^-2^. Thus obtained, PSi was mechanically pulverized in a mortar agate; then, the PSi powders were poured into different solutions to get the final Si-ncs colloidal solutions. The different optical, morphological, and structural characteristics of the colloidal solutions with Si-ncs were measured and studied. These Si-ncs colloidal solutions, measured by photoluminescence (PL), revealed efficient blue-green or violet emission intensities. The results of X-ray diffraction (XRD) indicate that the colloidal solutions are mainly composed of silicon nanocrystallites. The result of UV–vis transmittance indicates that the optical bandgap energies of the colloidal solutions varied from 2.3 to 3.5 eV for colloids prepared in methanol, ethanol, and acetone. The transmission electron microscopy (TEM) images showed the size of the nanocrystals in the colloidal solutions. Fourier transform infrared spectroscopy (FTIR) spectra showed different types of chemical bonds such as Si-O-Si, Si-CH_2_, and SiH_*x*_, as well as some kind of defects.

**PACS:**

61.46Df.-a; 61.43.Gt; 61.05.cp; 78.55.-m; 81.15.Gh

## Background

The research of nanoparticles with unique dimensionality dependent on the chemical-physical properties of nanoscale matter has propelled efforts towards controllable fabrication and in-depth characterization of inorganic nanostructures with desirable compositional and geometric features. At the forefront of the current scientific revolution of colloidal nanocrystals (C-nc), crystalline particles grown in liquid media stand out over other classes of inorganic nanomaterials due to the high degree of control with which their crystal structure, size, shape, and surface functionalities can be engineered in the synthesis stage and to the versatility with which they can be processed and implemented into a large number of materials and devices [1-3]. On the other hand, silicon is the semiconductor material predominant in the microelectronics industry. It has an indirect bandgap of 1.1 eV, which is the main limitation for application in optoelectronics and photonics [2], due to low probability of radiative transitions that make silicon a poor light emitter. However, unlike bulk silicon, silicon nanoparticles (silicon nanocrystals) have a behavior as a direct bandgap semiconductor [4,5]. After the discovery of visible light emission at room temperature in the porous silicon (PSi) by Canham [6] in 1990, many researchers have studied the emission properties of materials with Si nanocrystals (Si-ncs). Among many techniques to prepare Si-ncs, the electrochemical etching of porous Si remains the cheapest and fastest method; it may be the most promising for production of large amounts of Si-ncs required for potential applications in nanotechnology. Silicon-rich oxide (SRO) has shown photoemission. But it required normally a high-temperature annealing in order to get intense emission. Also, Si-ncs obtained by electrochemical methods and poured in solutions have shown photoemission that depends on their size. Thus, Si-ncs obtained by electrochemical methods are an alternative to obtain emission different to the ones of silicon-rich oxide but without a high-temperature annealing [7]. Colloidal dispersions containing monodispersed and polydispersed nanoscale particles are of great importance in the field of nanoscience and nanotechnology [8,9]. Their main characteristics are the well-defined dimensions and extraordinary functional properties [8,9], which have started to be used to elucidate the properties of colloidal matter [10]. In addition, particles with uniform structural properties are offering best properties for applications in different fields such as catalysis [11,12], gas sensors [13], biosensors [14], magnetic materials [15], and photonics [16] among others [17,18]. Significant efforts in this direction are being made to develop simple and reproducible methods capable of generating colloidal samples monodispersed and polydispersed in small and large quantities [8-10,19] based on colloidal chemistry [17,20]. The method we use for the synthesis of colloidal silicon nanocrystals (C-Si-ncs) is novel in many ways, because unlike other methods, this is easier to implement, fast, cheap, and has good control on the parameters used, unlike other well-known methods such as Stober, laser pulsed, controlled precipitation, emulsions, oxidation, combustion of silane, gas evaporation, and thermal degradation [21-34]. Also, in this work, we prove and present that using different solvents in the solutions results different behaviors, such as the change of color of the samples without the excitation. The photoluminescence (PL) (change in the emission wavelength) of the C-Si-ncs changes with the excitation wavelength, and this is another important optical property. Therefore, the goal of this work is to study and investigate the compositional and optical properties of C-Si-nc suspensions formed with pulverized porous Si layers, when the Si-nc powders are suspended in different organic solvents. Special attention is paid to a surprising observation of intense green and blue PL in C-Si-nc suspensions, which opens the possibility to propose novel applications in a future work.

## Methods

PSi powder has been prepared by standard electrochemical etching of Si wafers (<100 > p-type, *ρ* =0.01 to 0.02 Ω cm and diameter 2 in). Before the electrochemical etching, the wafers were cleaned in three different solutions (xylene, acetone, and methanol) for 11 min in ultrasonic bath and then the wafers were rinsed with deionized water. The solution to obtain PSi consisted of a mixture of peroxide of hydrogen/hydrofluoric acid (HF)/methanol (2:1:1).

The experimental method was realized as that of Valenta et al. [35]. We used an electrolytic cell of Teflon with a platinum electrode (cathode) and a brass electrode (anode), which has a current source, and then the solution was poured into the cell and a current in the source terminals is applied to the electrodes (anode and cathode) for the electrochemical reaction. The etching current density was varied to obtain a higher porosity and thickness. The current densities used to obtain the samples were varied from 250 mA cm^-2^, 1.0 A cm^-2^, 1.2 A cm^-2^, and 1.28 A cm^-2^. We used a short time (between 10 and 15 min) if a high-density current (>1.0 A cm^-2^) was used. Once the PSi layer was obtained, we rinsed it with deionized water to stop the chemical process. The samples obtained are listed in Table 1. After, the PSi films so obtained were scraped off the silicon wafer and subsequently milled in an agate mortar. The obtained powders were mixed together with the solvent selected. Then, we wait for around 2 weeks until the larger particles were decanted. This process can be accelerated using a centrifuge equipment. Colloidal solutions were prepared by pouring the powder in three different organic solvents (ethanol, methanol, and acetone). Finally, almost transparent solutions with intense green and blue PL under UV excitation were obtained. Such C-Si-nc suspensions are a starting point for production of various Si nanostructures with colloidal phase.

**Table 1:** Samples of C-Si-nc suspensions to different current densities and different solvents

**Sample**	**Current density (A cm**^-**2**^**)**	**Solvent**	**Porosity (%)**	**Thickness (μm)**
M-09	1.20	Methanol	70	12
M-10	1.28	Acetone	67	11
M-14	0.25	Ethanol	99	98
M-16	1.00	Acetone	96	41
M-17	1.00	Acetone	92	15
M-19	1.28	Acetone	65	8

The PL response was measured at room temperature using a Horiba Jobin Yvon spectrometer model FluoroMax 3 (Edison, NJ, USA) with a pulsed xenon excitation source and a multiplier tube detector controlled by a computer. The samples were excited using a radiation of 250 nm, and the PL response was recorded in the range from 400 to 900 nm with a resolution of 1 nm. Room-temperature transmittance spectra of the colloidal Si-nc suspensions were measured using an Cary 5000 UV–vis-NIR system (Agilent Technologies Inc., Santa Clara, CA, USA). The transmittance spectra were recorded in the range from 350 to 900 nm with a resolution of 0.5 nm. Fourier transform infrared spectroscopy (FTIR) was measured in the range from 450 to 4,000 cm^-1^ using a Bruker system model Vector 22 (Bruker Instruments, Billerica, MA, USA).

The X-ray diffraction (XRD) diffractograms were obtained with a D8 discover Bruker diffractometer operated at 40 kV and at 40 mA using the CuKα1 radiation (1.5406 Å). The powder with Si-ncs diffractograms were recorded to 2*θ*, which was varied from 5° to 80° in 0.02° step size (Bruker Instruments). The TEM images were obtained with JEOL-2010 HRTEM (Jeol, Tokyo, Japan), with a potential of 200 kV.

## Results and discussion

**Figure 1:**
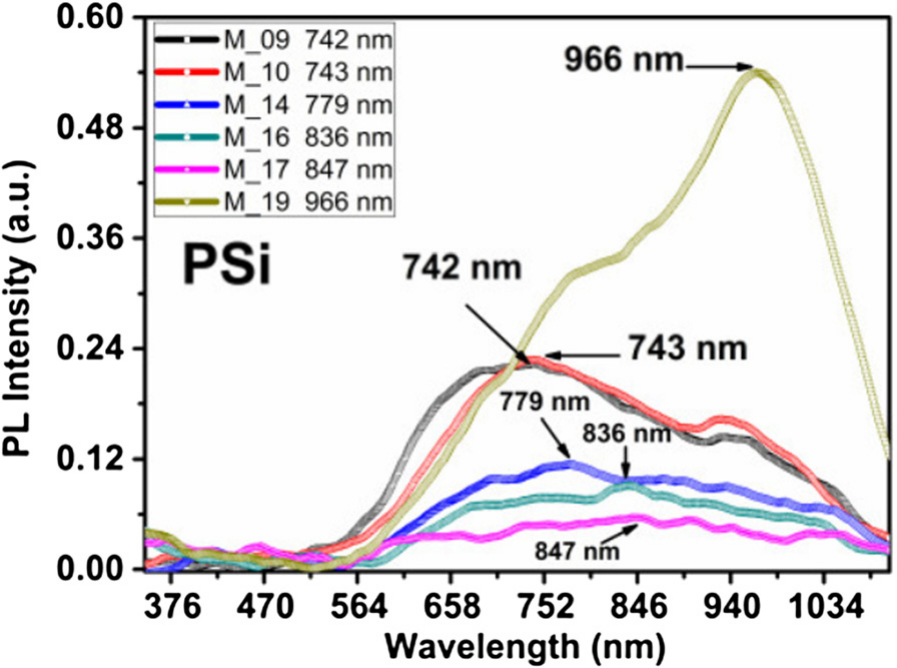
PL spectra of the PSi samples prepared with different etching current densities.

The PSi samples showed intense near-infrared PL spectra under UV excitation; all samples have a Gaussian-like shape with the maximum peak located between 743 and 966 nm, depending on the sample, as shown in Figure 1.

In Table 1, some preparation parameters of the PSi single layers and the C-Si-nc solutions are listed. Also, the results of porosity and thickness obtained by gravimetry [36] are listed. Below, we present the results and their discussion for the results with the different characterization techniques used.

### Photoluminescence

**Figure 2:**
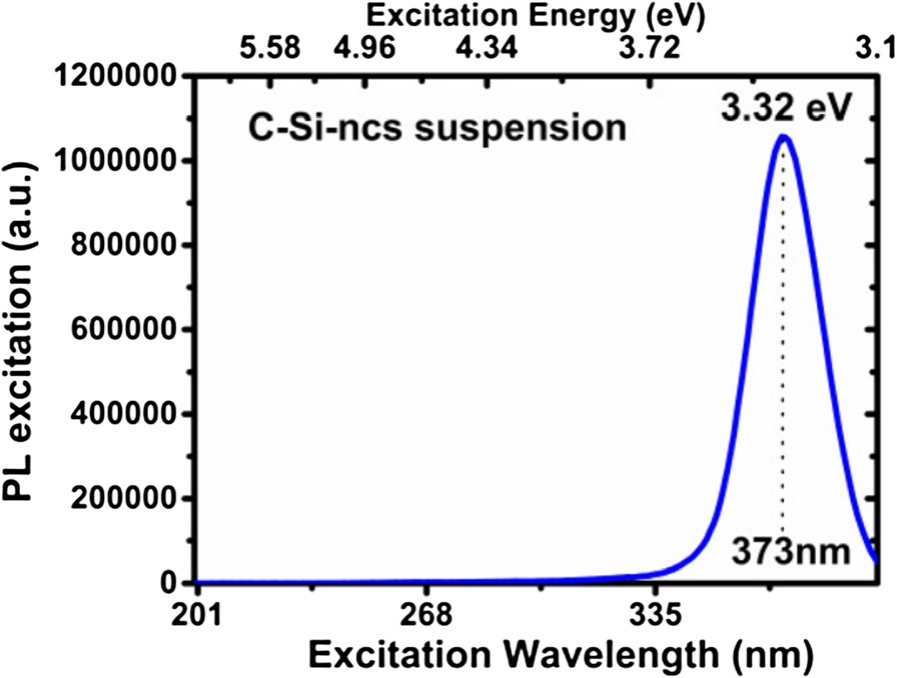
Excitation spectra of the C-Si-nc solutions with acetone as solvent.

**Figure 3:**
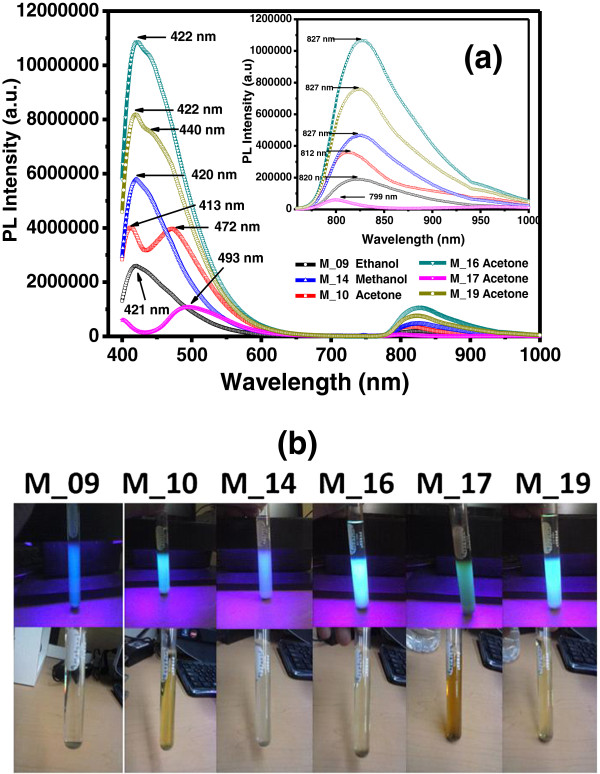
**PL spectra of C-Si-nc solutions and their respective colors under room light and UV excitation. (a)** PL spectra of C-Si-ncs solutions with different solvents. **(b)** Photographs of the C-Si-nc solutions under room light (lower part) and under UV excitation (upper part).

The C-Si-nc solutions with different solvents were first excited with different selected wavelengths from 200 to 400 nm to find the appropriate excitation wavelength. Then, the PL spectra were measured. Figure 2 shows an excitation spectrum of the C-Si-nc suspension with acetone as solvent.The C-Si-nc suspensions with different solvents show intense green, blue, and violet PL intensities, which depend on the solvent used. A similar PL behavior is observed in the infrared range, but in this case, the red PL intensity is smaller than the green-violet PL intensity, as shown in Figure 3a. It is important to note that based on the PL results, the process is repetitive; the similarity between the spectra of samples M-16 and M-19 and samples M-10 and M-17 confirms this. The colors of the C-Si-nc solutions are shown in Figure 3b. It is seen that M-16 is the most transparent solution and M-17 is the darkest solution. The PL intensity is related with this behavior. We can infer from this figure that the solution with the greatest PL intensity is the sample M-16.As can be seen in the inset of Figure 3a, the PL spectra have a Gaussian-like shape with the maximum peak position lying in the range from 799 nm to 827 nm. The peak position depends on the anodizing current density used to obtain PSi single layers and on the solvent used to obtain the C-Si-nc suspensions. However, it is not possible to observe a clear tendency of the PL intensity with respect to the current density and the solvent used.All colloidal solutions under UV light show bright green and violet PL emission. The solution M-09 shows an intense PL blue emission that can be observed by the naked eye, and this sample has methanol as solvent. The PL maximum peak is located at 421 nm, and its spectral width extends from 400 to 542 nm; also, it is observed in the spectrum that a secondary PL peak of low intensity is in the near-infrared range with a maximum at 820 nm as we can see in the inset of Figure 3a.The PL spectrum of the M-10 solution presents three peaks showing a PL maximum peak between green and violet. One peak is located at 413 nm; this wavelength is associated with the violet color and shows an intensity slightly greater than the peak located at 472 nm (second peak, associated with the blue color). The third peak shows a much lower intensity than the former peaks, which is located at 812 nm in the near-infrared region, see the inset in Figure 3a.

The PL spectrum of M-14 solution shows two peaks. The one with greater intensity is located at 420 nm and has a spectral range from 400 to 540 nm, and the other with much lower intensity is located at 827 nm.

The spectra of the M-16 and M-19 solutions which were prepared with the acetone as solvent are also shown in Figure 3. The peak with greater intensity is located at 422 nm and corresponds to a spectral range from 400 to 600 nm; and a secondary peak with much lower intensity is located at 827 nm. The PL spectra were obtained with 373-nm excitation radiation. In relation to this result, there are several similar studies that have been reported, such as that of Valenta et al. [35] which has a similar process as in this work, but the results obtained in their PL spectra in orange (like PSi) and PL spectra in green (after being filtered) were wider and with lower intensity due to the particle concentration in the solvent. In our experimental process, the PL intensities appear to be higher. Also, the results of Cortazar Martinez [7] show two peaks: one that is more intense in the blue region and the other in the red region [7], which is less intense. A similar result is reported by Nayfeh et al. [37] and Tilley et al. [38]. These results are due to the mechanical forces applied in an agate mortar to reduce the size of the PSi when it is milled together with the selected solvent. This affects the photoluminescence spectrum of the colloidal solution. Also depending on the applied forces, the Si-nc size differs, where the C-Si-nc suspensions change from transparent to dark, as can be see in Figure 3b. We obtain changes in the two PL peaks in the violet and near-infrared regions [37,38], where the stronger peak of violet PL dominates due to a larger amount of smaller size nanocrystals and the defects around the nanocrystals. Many models have been proposed to explain the PL such as quantum confinement [4,6,39,40], surface states [41], defects in the oxide [42], and even chemical species [43]. However, the definitive answer to these effects on PL [44] has not been found, but the accepted model is the quantum confinement with exceptions in the nanocrystal size from 1 to 5 nm [40,45]. However, it is difficult to exclude the presence of any thin oxide layer surrounding the Si-ncs which is necessary to saturate the dangling bonds on the surface or surrounding of the Si-ncs. Therefore, this is generally associated with defect states produced by the process of fabrication. Various authors in the literature have suggested the presence of defects in the samples and assigned an luminescent emission band around 2.7 eV from defect states at the silicon cluster-SiO_*x*_ interface; similarly, band emissions around 1.7 eV are also assigned to defects. On the other hand, the hydrogen bonds play an important role in the photoluminescence [46]. In our case, we have a lot of H bonds in the C-Si-nc suspensions structure, as is shown in FTIR spectra.

### Fourier transform infrared spectroscopy

**Figure 4:**
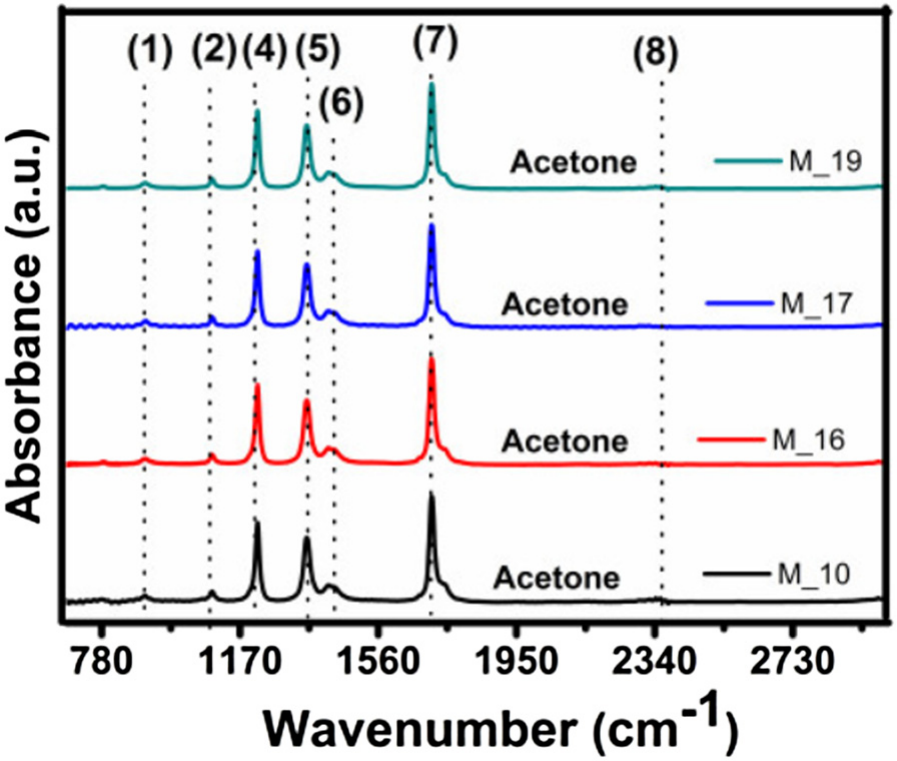
FTIR absorption spectra of C-Si-nc suspensions prepared with acetone as solvent.

**Figure 5:**
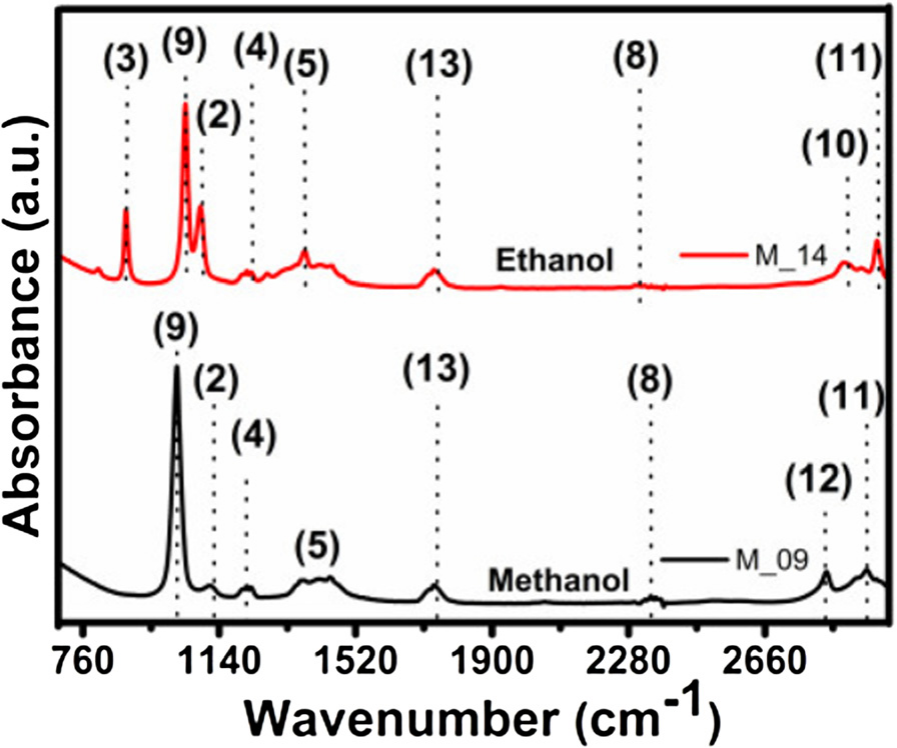
FTIR absorption spectra of C-Si-nc suspensions prepared with methanol and ethanol as solvents.

The FTIR spectra of the C-Si-nc solutions are shown in Figures 4 and 5. Different Si-O, Si-H, and C-H vibration modes are observed in the spectra of all the samples; but depending on the solvent used, their intensity and peak position change.

The solutions prepared in methanol and ethanol as solvents present additional peaks at around 800 and 1,260 cm^-1^. These peaks correspond to the vibration frequency of Si-(CH_3_)_*n*_ bonds. These bonds indicate that the methanol and ethanol molecules are decomposed during the strong milling of the PSi, which generate unbonded Si and CH_3_ molecules around the Si-ncs. Therefore, the possible origin of the strong PL intensity of methanol and ethanol suspensions is the Si-ncs related to the Si-(CH_3_)_*n*_ bonds. On the other hand, C-Si-nc suspensions with acetone show a stronger PL, and their FTIR peaks correspond to vibration frequencies different from those suspensions prepared with methanol as solvent, as shown in Figure 5.

The FTIR absorption spectra of the colloidal solutions are shown in Figure 5. The results of FTIR show different peaks of Si. For example, for the M-09 sample, the PL spectra show peaks at 1,113 cm^-1^ that correspond to Si-O-Si asymmetric stretching [46-49]. The PL spectra from the M-14 sample show peaks at 880 and 1,088 cm^-1^ that correspond to O_3_SiH bending [47,49] and Si-O-Si asymmetric stretching [46-49], respectively, and for the M-10, M-16, M-17, and M-19 samples, there are peaks found at 900 and 1,092 cm^-1^ that correspond to SiH_3_ degenerate deformation [46-49] and Si-O-Si asymmetric stretching [46-49], respectively. Other peaks appear from organic compounds such as CH_2_ asymmetric stretching at 2,800 cm^-1^[46] and at about 2,352 cm^-1^ that correspond to CO_2_ bonding [47].

The typical IR active vibrational modes of Si, Si oxide, and Si hydride are listed in Table 2 for comparison and clarity. The assignments of peaks in the FTIR spectra are made on the basis of these signature peaks. Before analyzing the features of interest, we identify the artifacts present in the FTIR spectra and ensure that they do not interfere with the analyses. It is seen that in all the spectra shown in Figures 4 and 5, there are peaks centered at about 2,350 cm^-1^ which is a typical signature of CO_2_[47]. Serial peaks in the range from 1,390 to 1,800 cm^-1^ accompanied with a noise-like signal are characteristic of ambient water vapor absorption and Si-CH_*n*_ in the samples [47,50]. These signals appear in all spectra due to the adsorption of IR active water vapor and CO_2_ molecules from the atmosphere. The intensity and spread (for the water vapor peaks) of these artifact IR active bands vary for the different samples, indicating different amounts of absorption during sample formation. As these peaks do not interfere with the peaks of interest, they are only considered as a reference. The FTIR spectra of the suspensions and their vibration mode assignments are listed in Table 2.

**Table 2:** FTIR spectra of C-Si-nc suspensions

**Peak number**	**Wavenumber (cm**^**-1**^**)**	**Group**	**Vibrational modes**	**Reference**
1	900	SiH_3_	Degenerate deformation	[46,47,49]
2	1,113, 1,080, 1,092	Si-O-Si in SiO_2_	Stretching	[46-49]
3	880	O_3_SiH in SiO_2_	Bending	[47,49]
4	1,200 to 1,236	Si-CH_3_ or Si-CH_2_	Symmetric bending	[50]
5	1,360	C-H	Bending	[50]
6	1,390 to 1,460	C-CH_3_ or SiCH_3 or 2_	Deformation or scissoring	[50]
7	1,710	Carboxyl		[48]
8	2,350	CO_2_	Room ambience	[47]
9	1,020	Si-OR	Stretching	[50]
10	2,880	C-CH_2 or 3_	Stretching	[50,51]
11	2,974	C-CH_3_ or Si-CH_3_	Asymmetric stretching	[50]
12	2,820	C-CH_2_	Symmetric stretching	[50]
13	1,740	Ambient water vapor		[47]

Peak positions of FTIR vibrational modes and their assignment.

For the nanostructures with C-Si-ncs treated with acetone as solvents, different bands extending from approximately 900 to 1,260 cm^-1^ are present. These bands (1, 2, and 9) do not have high absorbance, only the peak 4 which correspond to Si-H_3_, Si-CH_*n*_, SiOR, and Si-O-Si, respectively. There is a very small peak around 800 cm^-1^ which is assigned to an O_3_SiH bond in SiO_*x*_. A very weak peak seems to appear at about 2,110 cm^-1^, but it is difficult to distinguish unless compared with the other sample spectra.

For the nanostructures with C-Si-nc treated with methanol and ethanol as solvents, different bands extending from approximately 900 to 1,260 cm^-1^ are present, with a distinct peak at 1,080 cm^-1^ (there is also an appearance of a shoulder at approximately 1,195 cm^-1^). HF etched Si-ncs in PSi; therefore, the spread of the band with a peak at approximately 1,080 cm^-1^ is the same as that of C-Si-ncs with acetone as solvent; similarly, another broad band with a peak at about 880 cm^-1^ is identified.

The FTIR spectra clearly show the presence of oxygen in all the samples. The broad band at around 1,080 cm^-1^ is assigned to the Si-O-Si bond stretching. Pai et al. [52] and Lucovsky et al. [53] have shown that the position of the principal absorption band, i.e., the Si-O-Si stretching band of the Si oxide, is sensitive to the O-to-Si ratio. According to these authors, the peak of the Si-O-Si stretching band at 1,080 cm^-1^ corresponds to non-stoichiometric SiO_*x*_. Therefore, it can be concluded that SiO_*x*_ is present in all samples.

This reduced sharpness of the distinction between the peak and the shoulder supports the non-stoichiometry of the Si-O-Si stretching band [53]. The origin of hydrogen in the PSi-etched Si-ncs in the form of Si-H bonds is in all possibility from the trapped moisture since the enormous surface area adsorbs moisture when exposed to the ambient atmosphere. Thus, the presence of hydrogen species can tentatively be assigned to originate from the adsorbed PSi on the surface which formed hydrogen bonds during the etching process. The samples are dominated by oxygen, too. This observation implies that the etching process replaces oxygen with hydrogen, and the hydrogen passivation is greater for HF. Although the presence of the Si-O-Si bands are also clearly detected in the C-Si-nc suspensions, it is difficult to conclude that these are residual oxides due to the exposure of the Si-ncs to the ambient atmosphere. Pi et al. [54] observed that Si-ncs get passivated exclusively by hydrogen immediately after HF etching, but signatures of Si-O-Si grow with time as the Si-ncs are exposed to the atmosphere. This is because hydrogen does not completely passivate defects such as dangling bonds at the surface of the C-Si-ncs. Then, there is a subsequent passivation by oxygen. The increase in intensity of the Si-O-Si bonding peaks at approximately 800, 1,080, and 1,113 cm^-1^ on the oxidized Si-ncs is probably due to oxygen capture from the atmosphere by the enhanced defect centers. Therefore, the FTIR results suggest that all C-Si-nc suspensions are capable of creating isolated Si-ncs and passivating Si with hydrogen, but at the same time, creates an increase of defects at the surface. The defects at the surface increase, which are subsequently passivated with oxygen on exposure to ambient atmosphere.

### UV–vis spectroscopy

**Figure 6:**
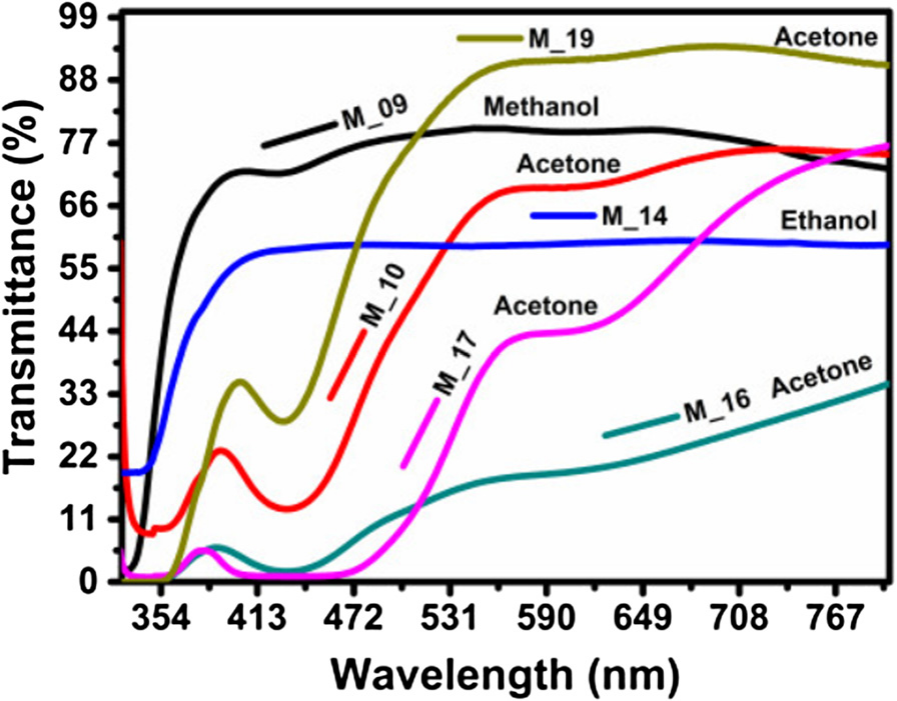
UV–vis transmittance spectra of C-Si-ncs suspensions prepared with acetone, methanol, and ethanol as solvents.

The optical bandgap of the C-Si-ncs suspensions is obtained from UV–vis spectra measurements. Transmittance spectra for C-Si-nc suspensions are shown in Figure 6. The transmittance spectra of the C-Si-nc solutions with acetone as solvent are relatively high (90%) from 550 to 800 nm, as shown in Figure 6. The transmittance spectra of the C-Si-nc suspensions with methanol and ethanol are smaller than the ones obtained for samples with acetone as solvent. The Si-nc size with the different organic solvents produces a clear change in the form of the spectra and a shift towards lower wavelengths. Also, in these transmittance spectra, it is possible to observe that some curves have two or three oscillations from 300 to 600 nm; therefore, two possible regions of strong-medium absorption are present. These absorptions can be contributions of different band-to-band transitions for extended states or localized states, as will be discussed later.

To obtain the bandgap of our solutions, we use the method of inflection point. This method is based on the minimum photon energy needed to promote electron–hole pairs. This point given to a certain wavelength indicates whether the absorption of photons is sufficient to generate electron transitions from the valence band to the conduction band; if not, these photons have a lower band-gap of the material energy. Mathematically, the inflection point is located when the second derivative is zero or does not exists. In our case, with the first derivative, it showed maximum peaks and when applied to the second derivative, these peaks are zero. But with the first derivative, it is easier; see details and the obtained bandgap energy reported in [47]. Now, the M-09 sample represents a peak at 349 nm that corresponds to an optical energy bandgap of 3.55 eV. For the M-14 sample, the point of inflection is located at 357 nm, and it corresponds to optical energy bandgap of 3.47 eV. Now, for the M-10, M-16, M-17, and M-19 samples, the derivative presents two or three peaks at different energies. For example, in M-10 and M-16, the first peaks are located at 371 and 369 nm, respectively; the second peaks are at 475 and 469 nm, in energy terms correspond to 3.34 and 3.36 eV, and 2.61 and 2.64 eV, respectively. The M-17 and M-19 samples have the first peaks at 350 and 380 nm, the second peaks at 450 and 580 nm, and the third peaks at 600 and 775 nm, which correspond to 3.54 and 3.26 eV, 2.75 and 2.13 eV, and 2.06 and 1.6 eV, respectively.

The different peaks of the derivative of transmittance spectra that have been found have been attributed to smaller particles in the colloidal solutions as in the silver colloidal solutions [55]. Other authors have reported the same effect, but they do not say anything about these effects [50,56]. Also, the behavior of the transmittance spectra of the colloidal solutions with different solvents change depending of the solvent used [57]; in this reference, they used other method of synthesis, and the bandgap obtained in this case is in the range from 2.4 to 3.2 eV [57]. These results are similar to the ones obtained in this work, approximately 2.3 to 3.3 eV. On the other hand, according to the results here obtained, and in the case of the C-Si-nc suspensions, the electron density of states is likely to decrease with decreasing size of the Si-ncs. Then, a decrease of the absorption coefficient of Si-ncs can be understood [58]. Probably, the position of the derivative peaks can be related to the average size and distribution of Si-nc, where the position and size of the Si-ncs are dispersed, resulting in a change of the density of states due to bonds that correspond to Si-H_*n*_, Si-O, Si-CH_*n*_, and Si-OR.

**Figure 7:**
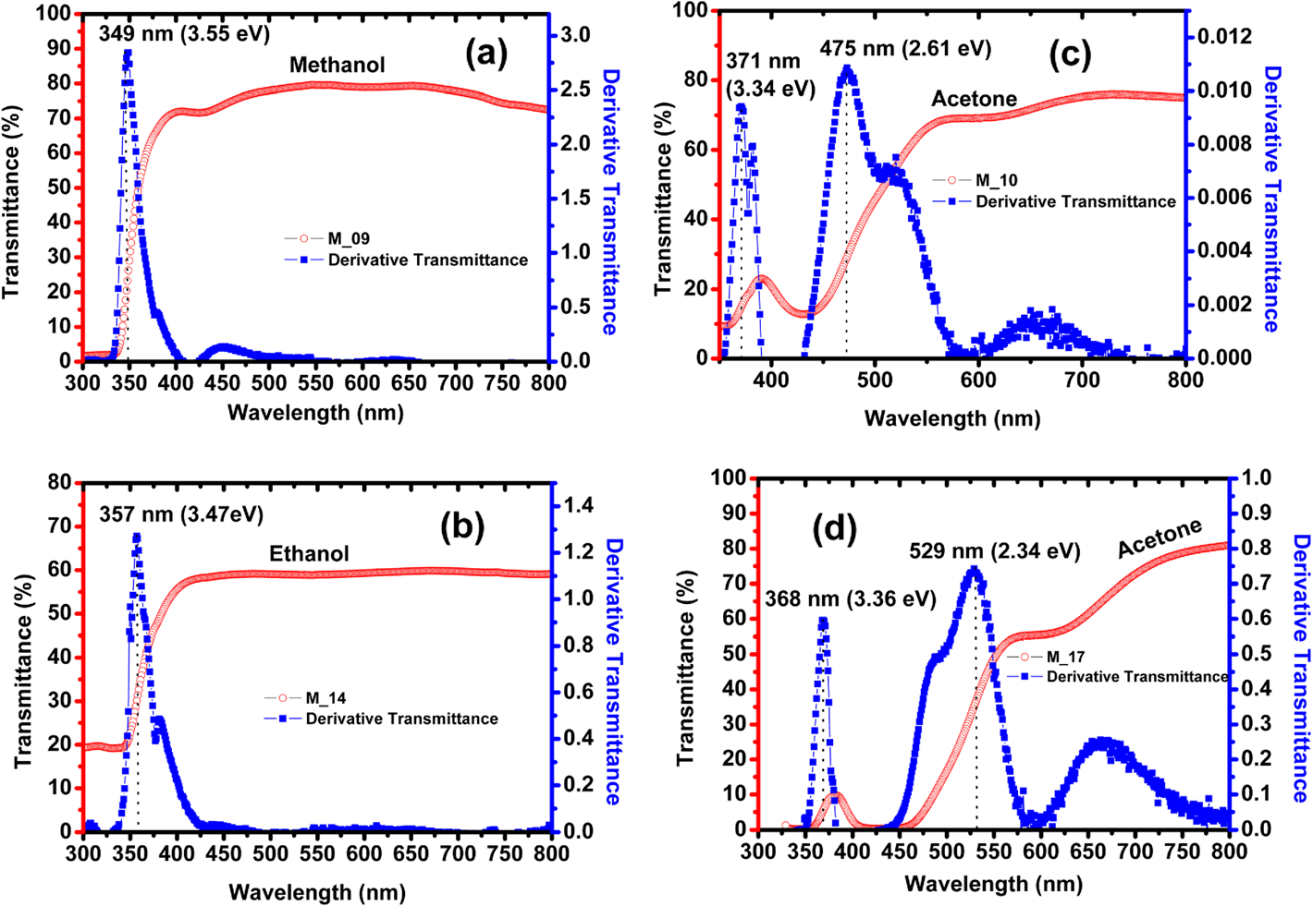
**UV–vis transmittance spectra of C-Si-nc suspensions and derivative of transmittance spectra to obtain optical bandgap. (a)** Colloidal suspension with methanol as solvent. **(b)** Colloidal suspension with solvent ethanol as solvent. **(c and d)** Colloidal suspension with acetone as solvent.

In Figure 7, it appears that the derivative peak is dependent on the solvent, average size, and distribution of Si-nc in the range from 1.8 to 3.5 eV, where it is clear that the absorption is due to indirect transitions side to side because the first direct band (3.2 eV) is outside this range. In this case, due to the different derivative peaks, we can obtain the different optical bandgaps in only one sample and then there are different mechanical transitions. Kovalev et al. [59] found that PSi absorption is of exponential behavior in the range from 1.5 to 3.0 eV. Therefore, we considered similar C-Si-nc suspensions and propose that this is due to the absorption in the surface states and also to phonon-assisted optical absorption as expected from the indirect nature of c-Si but also direct transitions because Si-ncs are expected. In our case, we can attribute the transmittance derivative peaks' nature of the structure to the indirect and direct absorption bands because there is a fundamental connection between absorption and indirect and direct band structures of the Si-ncs and defect.

From the results of photoluminescence, the PL spectrum extends below the absorption edge of the Si-ncs (3.2 eV). Therefore, the existence of forms and Stokes photons above the bandgap indicates that the radiative recombination takes place not only between shallow trapped electron and hole states [39] but also between quantized states of electrons and holes. Some of the electrons within the nanocrystals absorb photons and relax to localized states by phonon emission or emission photon and then there recombined with holes radiatively, while some of the electrons absorb photons and directly recombine radiatively without any phonon emission to the nanocrystals. Furthermore, theoretical calculations show that the size of the nanocrystals reduces the possibility of transitions involving phonon, increasing due to the quantum size effect, where Si-ncs are direct band structures. Therefore, the Si-ncs and defects (as shallow traps, surface states, and localized states) always take part in the process of luminescence or absorption in the Si-ncs-defects system.

### X-ray diffraction

**Figure 8:**
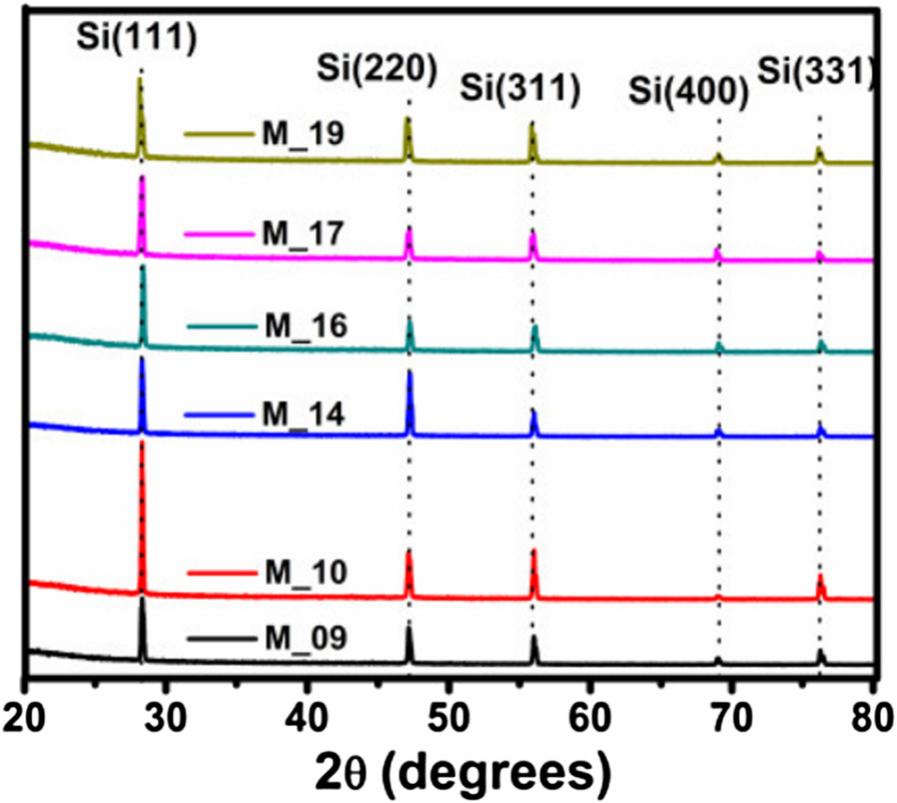
XRD spectra of the Si-nc powder obtained from PSi synthetized with different current densities.

The XRD patterns of the Si-nc powders through PSi were obtained and presented in Figure 8. We suppose that the changes in the peak intensities and peak position indicate that the concentration and size of the Si-ncs depend on the current density used to obtain the PSi layers.

**Figure 9:**
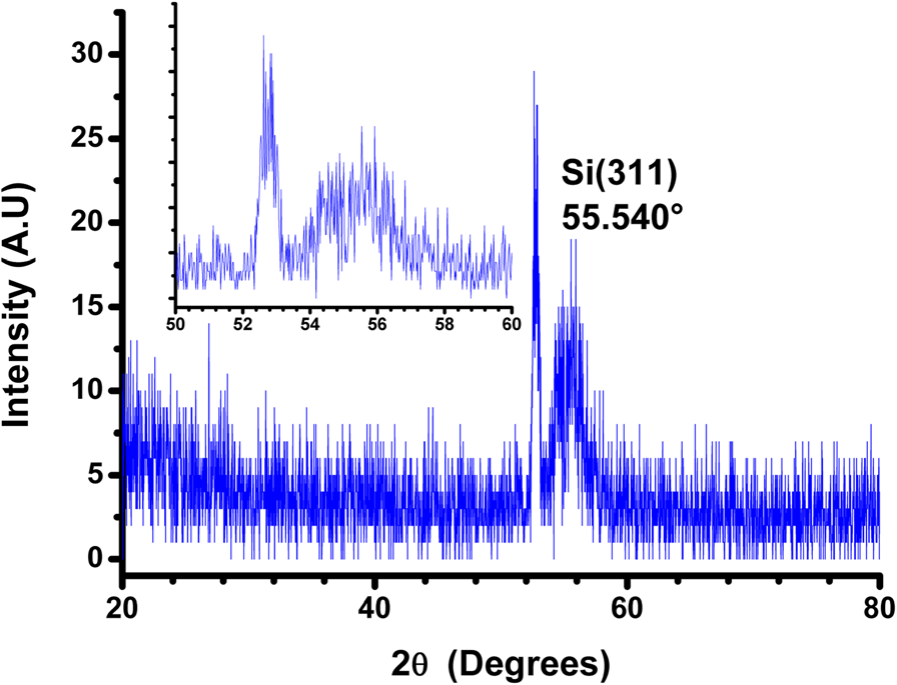
**XRD spectra of the C-Si-ncs with acetone.** Inset shows an approximation of the peak Si (311).

All peaks present in the diffractograms are located close to peaks in [60] and also close to crystal planes in [47,60,61]. This indicates that Si-ncs exist in our powders. XRD data prove that these films consist of Si-ncs with preferential (111) orientation. The small additional peaks around 2*θ* = 47.193° (220), 56.023° (311), 68.989° (400), and 76.261° (331) can be connected with some metastable Si states. Using the full width at half maximum (FWHM) of the peaks around 28.346° (111), 47.193° (220), 56.023° (311), 68.989° (400), and 76.261° (331), it was possible to calculate the average size of the Si-ncs by using the Scherrer equation [62,63]. Table 3 shows a comparison between the diffraction peaks of the JCPDS card 27–1402 and those of [60], with respect to the peaks obtained from Si-nc powders in this work. The intensity of the XRD signals, which is proportional to the volume of the Si-ncs, changes with the increase of current density for five types of Si-ncs.With the XRD spectra of Figure 9, it was found that our colloidal solutions have nanocrystals with average sizes of approximately of 6 nm and corroborated with the TEM image in the next section.

**Table 3:** Typical XRD diffraction peaks of the Si-nc powders obtained by PSi with different current densities

**Ref. [**[47]**,**[60]**] peak position (PP)**	**Sample M-09**		**Sample M-10**		**Sample M-14**		**Sample M-16**		**Sample M-17**		**Sample M-19**	
	**PP**	**Size (nm)**	**PP**	**Size (nm)**	**PP**	**Size (nm)**	**PP**	**Size (nm)**	**PP**	**Size (nm)**	**PP**	**Size (nm)**
28.44	28.34	41.57	28.33	98.63	28.33	61.83	28.41	72.43	28.32	43.30	28.18	52.22
47.30	47.19	44.75	47.19	42.53	47.24	60.37	47.25	41.64	47.24	39.34	47.06	32.20
56.12	56.02	50.28	56.02	122.7	56.02	56.10	56.08	38.19	55.99	37.48	55.85	56.9
69.13	68.98	48.35	68.97	39.10	68.12	32.75	69.06	80.54	68.91	60.07	69.06	99.88
76.38	76.26	44.5	76.26	40.27	76.27	39.26	76.31	38.16	76.17	44.05	76.13	48.98

The different planes of Si are indexed according to JCPDS card 27–1402.

### TEM images

**Figure 10:**
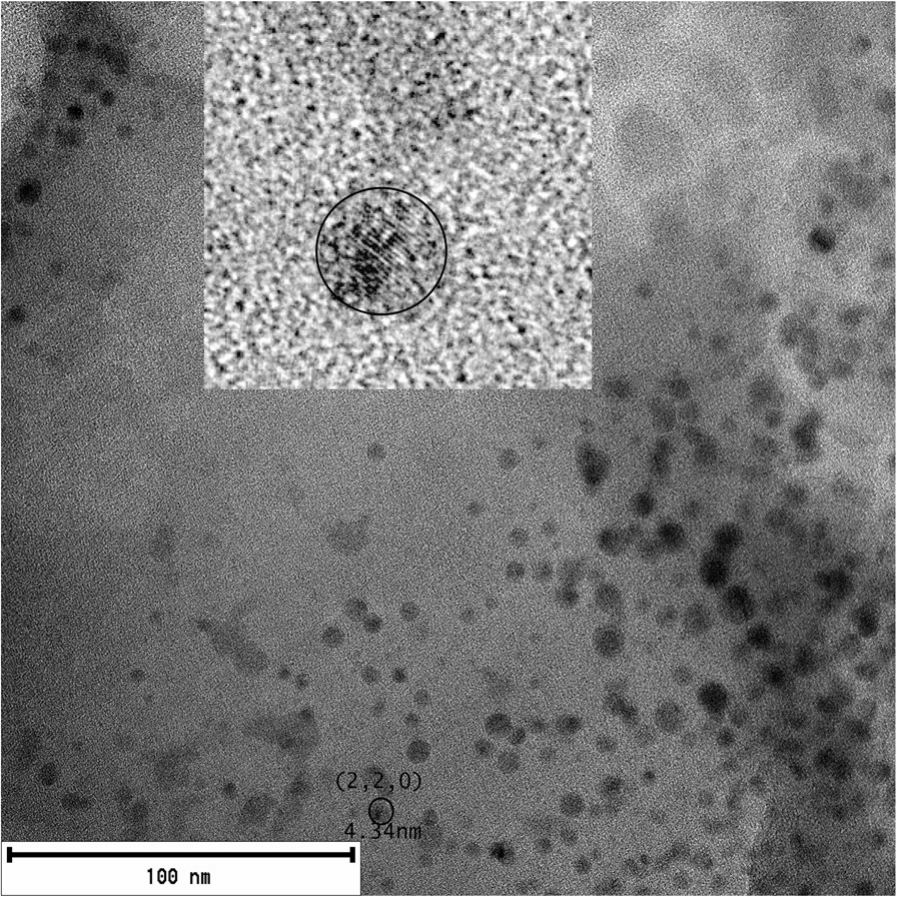
**TEM image of the C-Si-ncs with acetone.** Inset with the encircled area shows an approximation of a Si-nc.

With XRD, we find that the silicon nanocrystals with an average size of approximately 6 nm have similar sizes with those obtained with TEM images (4.34 nm). These results are similar to those reported by Ray et al. [47]. The size in the colloidal solutions based in XRD and TEM is between 4 to 6 nm. This shows that we have different sizes in our samples and our systems are polydispersed (Figure 10).

Finally, other similar works with all these characterizations have been reported by Ray et al. [47]. Their synthesis by controlled oxidation of mechanically milled silicon is an extremely slow process (100 h). The characterization realized in this work is very similar to ours. For example, in XRD, they get three peaks and nanocrystals between 14 to 30 nm in samples of powders. Similar size is obtained in our powders. TEM images show sizes of nanocrystals between 2 to 6 nm, and in our results, the size of C-Si-ncs is between 4 and 6 nm. On the other hand, with respect to FTIR, several groups with silicon oxide, silicon-hydrogen, and carbon-hydrogen bonds were obtained. Their PL spectra have some resemblance with ours because they also had double peaks of photoluminescence in the red region. Similar behavior is obtained in the UV–vis spectra. The bandgap was calculated using the same method, with energies of the same order as our results. This demonstrates that there is a method simpler to get results like that of Ray et al. [47]. Regarding the working life of the colloidal solutions, our samples were synthesized more than 6 months ago and are still presenting the same intense photoluminescence. Even when the solvent evaporates, depositing the same solvent would result to the same PL. These materials have a lot of application because they exhibit unique physical and electronic properties which can be used as substitutes of SRO films, such as thin film to devices, where the C-Si-ncs can be used to different applications such as photovoltaic, photodetectors, and emission light.

## Conclusions

In summary, in this study, the process was performed to remove the luminescent PSi nanostructures by scraping the PSi from electrochemically etched silicon wafer. The obtained powders were milled together with a selected solvent, and colloidal suspensions with silicon nanocrystals (C-Si-ncs) were obtained. On the other hand, HF etching is capable of producing free-standing isolated spherical quantum dots of Si with nanometric dimensions. The FTIR study revealed that along with the creation of isolated Si-ncs and hydrogen passivated Si, an increment of defects is also introduced at the surface of the Si-ncs; these defects are subsequently passivated by oxygen on exposure to ambient atmosphere. The PL characteristics of the C-Si-nc suspensions of these nanostructures are characterized by double peaks, and the observations provide direct evidence for quantum confinement-induced widened bandgap transitions and oxide-related interface state-mediated transitions and also allow us to experimentally distinguish between the contributions of the two transition mechanisms. Therefore, the PL emission can be correlated with quantum effects and defects in C-Si-nc suspensions, where also the absorption FTIR spectra show peaks associated with Si compounds. Those are also associated with defects. Then, a relationship between the composition and PL was obtained. Also, the different Si-nc sizes and organic solvents of the C-Si-nc suspensions produce a clear change of the transmittance spectra and a shift towards lower wavelengths.

## Competing interests

The authors declare that they have no competing interests.

## Authors’ contributions

JALL and AGR participated in the growth of the films; carried out the FTIR, photoluminescence, and UV–vis measurements; and drafted the manuscript. JFFG and JCL conducted the photoluminescence measurements. EGB and JMJ conducted the XRD measurements. AGR coordinated the study. JALL provided the idea and supervised the study. All authors read and approved the final manuscript.

## Authors’ information

JALL is currently a researcher and professor in the Science Institute-Center of Investigation for Semiconductors Devices (IC-CIDS) from Autonomous University of Puebla, Mexico. He has been working on structural, electrical, and optical characterization of materials and MOS structures. His research interest is the physics and technology of material nanostructures and silicon devices. Additionally, his research interests are nanotechnology, material characterization, and optoelectronic devices such as sensor, LEDs, and solar cells. EGB is a researcher and instructor in the Research Center of Semiconductor Devices of the Institute of Science, Benemérita Universidad Autónoma de Puebla, México. She has been working mainly on the synthesis of semiconductor materials and their optic, morphologic, and photocatalytic properties, and their composition. Her actual research interest is on the synthesis, characterization, and applications of porous silicon.
